# A Cluster Randomized Trial of an mHealth Intervention for Adolescents With Congenital Heart Disease: Rationale and Design of the READYorNot CHD Study

**DOI:** 10.1016/j.cjcpc.2025.06.001

**Published:** 2025-06-07

**Authors:** Andrew S. Mackie, Adrienne H. Kovacs, Daniella San Martin-Feeney, Alicia Via-Dufresne Ley, Brian W. McCrindle, Kevin C. Harris, Anne Fournier, Alyssa Chappell, Jody Gingrich, Gina Dimitropoulos, Brooke Allemang, Navreet Gill, Sandra Aiello, Martha Rolland, Sunita O’Shea, Lea Legge, Jennifer A. Collins, Susan Casey, Fabiola Breault, Frederique Provencher, Rebecca Balmir, Rocio Gutierrez Rojas, Maryna Yaskina, Scott Klarenbach, Jennifer Zwicker, James M. Brophy, Roberta L. Woodgate, Ariane J. Marelli

**Affiliations:** a Department of Pediatrics, University of Alberta, Edmonton, Alberta, Canada; b Division of Cardiology, Stollery Children’s Hospital, Edmonton, Alberta, Canada; c Equilibria Psychological Health, Toronto, Ontario, Canada; d McGilll Adult Unit for Congenital Heart Disease Excellence, Montreal, Québec, Canada; e Division of Cardiology, The Hospital for Sick Children, Toronto, Ontario, Canada; f Division of Cardiology, British Columbia Children’s Hospital, Vancouver, British Columbia, Canada; g Division of Cardiology, Centre Hospitalier Universitaire Ste-Justine, Montreal, Québec, Canada; h Faculty of Social Work, University of Calgary, Calgary, Alberta, Canada; i Women and Children’s Health Research Institute, Edmonton, Alberta, Canada; j Department of Medicine, University of Alberta, Edmonton, Alberta, Canada; k School of Public Policy, University of Calgary, Calgary, Alberta, Canada; l Department of Medicine, McGill University, Montreal, Québec, Canada; m Rady Faculty of Health Sciences, College of Nursing, University of Manitoba, Winnipeg, Manitoba, Canada

**Keywords:** clinical trial, adolescent, congenital heart disease, education, continuity of care, transition of care, mobile Health, electronic Health

## Abstract

The population of adolescents with congenital heart disease (CHD) is growing exponentially and requires transition preparation for adult-oriented health care. Nurse-led transition programs are effective in improving CHD knowledge and self-management skills. However, many clinical programs lack the human resources needed to provide transition services. Mobile health applications have the potential to prepare transition-age youth for entering adult health care. However, there are no outcome data on the impact and effectiveness of CHD transition applications. Accordingly, in partnership with a Youth Advisory Council, we developed the MyREADY Transition CHD App (the App) designed to enhance youth CHD knowledge and self-management skills. The READYorNot CHD study is a multicenter, cluster randomized noninferiority clinical trial that is evaluating the efficacy of the App plus limited nurse teaching (intervention), vs comprehensive nurse-only teaching (control) for 16- to 17-year-olds with moderate or complex CHD. Participants are being enrolled in clusters based on week of attendance in the pediatric cardiology clinic, with a 1:1 allocation between intervention vs control and target recruitment of 204 participants. The primary outcome is the change in Transition Readiness Assessment Questionnaire score from baseline to 18 months. Secondary outcomes are change in CHD knowledge score, self-efficacy, and time to first adult CHD appointment. Semistructured interviews will provide additional insights into the advantages and disadvantages of the App vs nurse-only teaching. This study will inform patients, pediatric cardiology programs, and policy makers in judging whether this mobile health intervention warrants widespread availability in clinical settings to improve transition outcomes of adolescents with CHD. Clinical Trial Registration: NCT04463446.

## Résumé

La population des adolescents qui vivent avec une cardiopathie congénitale (CC) connaît une croissance exponentielle, et une préparation à la transition vers les soins destinés aux adultes est nécessaire pour ces patients. Les programmes de transitions menés par le personnel infirmier constituent une façon efficace d’améliorer les connaissances sur la CC et les capacités d’autoprise en charge. Plusieurs programmes cliniques ne disposent toutefois pas des ressources humaines nécessaires pour offrir des services de transition. Les applications mobiles de santé pourraient permettre de préparer la transition des jeunes qui passent à des soins destinés aux adultes, mais il n’existe aucune donnée sur les retombées ni sur l’efficacité des applications pour la transition des patients atteints de CC. En partenariat avec un conseil consultatif de jeunes, nous avons donc développé l’application MyREADY Transition CHD App, une application spécialement conçue pour améliorer les connaissances et les capacités d’autoprise en charge des jeunes qui présentent une CC. L’étude READYorNot CHD est un essai clinique de non-infériorité multicentrique avec répartition aléatoire par grappes évaluant l’efficacité de cette application, en appoint à une courte formation par le personnel infirmier (groupe d’intervention), comparativement à une formation complète par le personnel infirmier (groupe témoin) chez des patients de 16 à 17 ans présentant une CC modérée ou complexe. Les participants sont répartis par grappes selon la semaine de leur présence en clinique de cardiologie pédiatrique, avec une répartition selon un rapport 1:1 entre le groupe d’intervention et le groupe témoin; l’objectif de recrutement s’élève à 204 participants. Le critère d’évaluation principal est le changement du score obtenu au Transition Readiness Assessment Questionnaire entre le début et 18 mois plus tard. Les critères d’évaluation secondaires sont les changements du score à une évaluation des connaissances sur la CC et du score à une évaluation du sentiment d’auto-efficacité et le temps écoulé avant le premier rendez-vous en soins pour adultes atteints d’une CC. Des entretiens semi-dirigés permettront d’obtenir de plus amples renseignements sur les avantages et les inconvénients de l’utilisation d’une application par rapport à des formations données uniquement par le personnel infirmier. Cette étude pourra guider les patients, les programmes de cardiologie pédiatrique et les décideurs dans la prise de décision sur une éventuelle diffusion plus large de cette intervention par une application mobile de santé en contexte clinique afin de favoriser une meilleure transition vers l’âge adulte chez les adolescents vivant avec la CC. No d’enregistrement de l’essai clinique : NCT04463446.

The number of adults with congenital heart disease (CHD) now significantly exceeds the number of children, reflecting decades of success in pediatric CHD care.^1^ This survivor population has complex needs requiring lifelong specialized CHD follow-up. Accordingly, preparing youth for the transition from pediatric to adult CHD (ACHD) care has become a major focus in the past decade. Numerous guidelines and position statements have been published to promote improved quality of care in transition processes.^2^, ^3^, ^4^ However, there remains a paucity of outcome data regarding the impact and effectiveness of CHD transition interventions. Furthermore, early adulthood is a life stage associated with lapses in care,^5^ reflecting a failure of transition preparation. Indeed, there is a lack of high-quality transition outcome data for pediatric-onset chronic health conditions.

Our team previously designed and conducted the **C**ongenital **H**eart **A**dolescents **P**articipating in **T**ransition **E**valuation **R**esearch (CHAPTER 1) study,^6^ followed by the CHAPTER 2^7^ and 3^8^ studies; these trials evaluated nurse-led transition education interventions, focusing on CHD knowledge^6^, ^7^, ^8^ and self-management skills^7^ among adolescents. These studies demonstrated that nurse-led teaching significantly improves patients’ knowledge of their CHD and self-management skills. However, nurse-led transition teaching is resource-intensive, requiring nursing time and expertise as well as space in clinical settings that may not be readily available. Nurse-led teaching may also be less accessible to patients living remote from cardiac centers, as increased distance between home and hospital has been associated with lapses in care.^9^ Furthermore, patients living in remote communities are more likely to be Indigenous^10^ and of lower socioeconomic status^11^ and may face financial barriers to attending in-person visits.^12^ Mobile health applications (apps) have the potential to mitigate some of these barriers and provide a more convenient resource to young adults as they enter adult care. Accordingly, the current study was designed to build on our experience with the CHAPTER studies by replicating the content of nurse-led teaching into an app that is youth-oriented and youth-informed.

The objectives of the READYorNot CHD study are to compare the impact of a smartphone app combined with limited nurse teaching, vs comprehensive nurse-only transition teaching, on transition readiness, CHD knowledge, self-efficacy, and time to ACHD care among youth graduating from pediatric cardiology programs. We hypothesize that the MyREADY Transition CHD App (the App) will not be significantly worse than the standard of care (ie, comprehensive nurse-only teaching) with respect to transition readiness, CHD knowledge, self-efficacy scores, and time to first ACHD clinic appointment. Semistructured interviews of participants in both groups will provide additional insights into the acceptability and merits of the App vs nurse-only preparation. This article will describe the methods of the proposed study.

## Methods

### Study design

**Figure 1:**
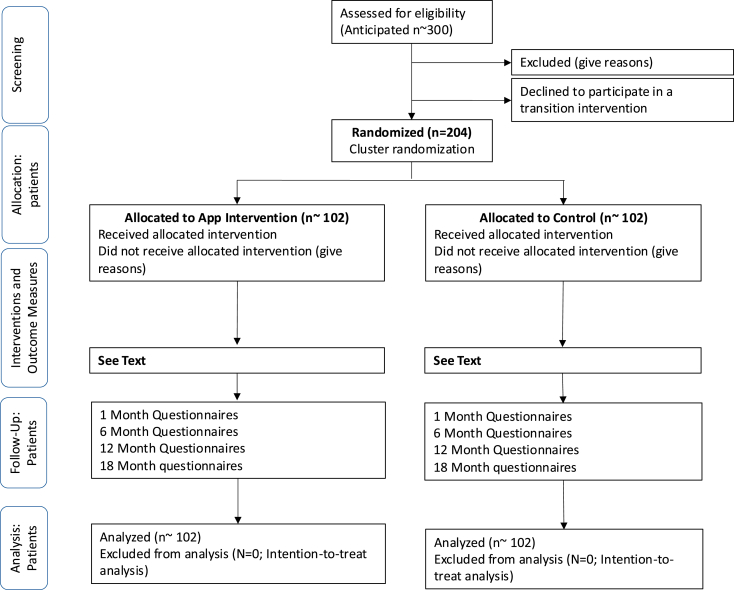
CONSORT (Consolidated Standards of Reporting Trials) diagram.

The READYorNot CHD study is a multicenter, cluster randomized controlled trial comparing access to the App plus limited nurse teaching (intervention group), vs standard-of care comprehensive nurse-only transition teaching (control group) in a 1:1 allocation ratio. The study is registered with Clinical Trials.gov (NCT04463446) and is being conducted in accordance with CONSORT (Consolidated Standards of Reporting Trials) guidelines (Fig. 1).

### Study setting and participants

**Figure 2:**
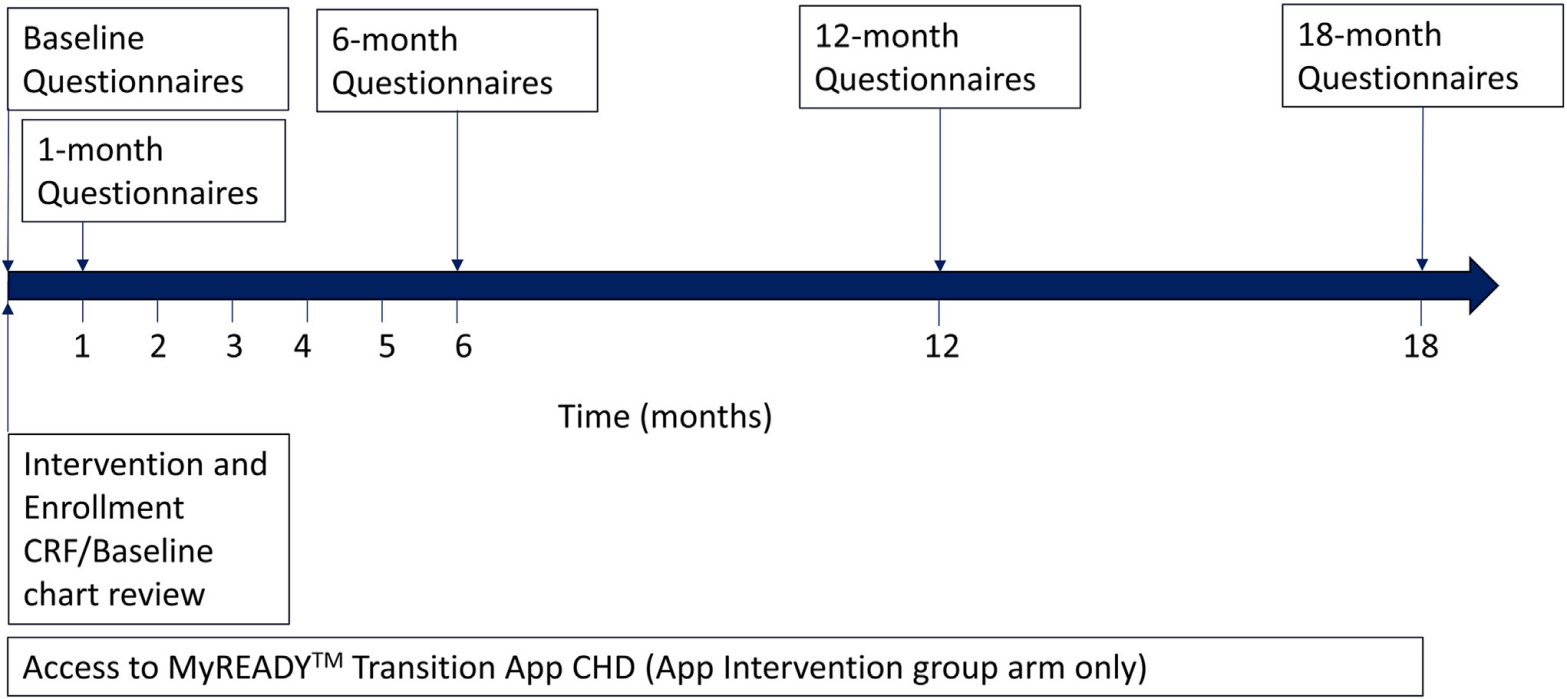
Individual participant timeline. CHD, congenital heart disease; CRF, case report form.

The study is being conducted at the Stollery Children’s Hospital (Edmonton, Canada), the Hospital for Sick Children (“SickKids,” Toronto, Canada), British Columbia Children’s Hospital (Vancouver, Canada), and the Centre Hospitalier Universitaire Sainte-Justine (Montreal, Canada). These are the largest pediatric cardiology programs in Canada and offer the full range of cardiology and cardiovascular surgical services. Inclusion criteria are 16- to 17-year-olds with moderate or complex CHD (as previously defined)^13^ who have not yet transferred to adult care. Although transition interventions should begin typically by 12 years of age,^2^^,^^3^ we chose to focus on older adolescents. Transition interventions need to be consistent with the developmental stage of participants, and it is not practical to develop an app that addresses the needs of the full range of typical 12- to 17-year-olds. Furthermore, it would be difficult to conduct a study that compares 2 transition interventions spanning 5-6 years each (ie, from ages 12 to 17 years). Exclusion criteria are (1) less than a grade 6 level of reading and comprehension, based on medical records review or parent report, or (2) a history of heart transplantation, as this results in distinct health challenges. To prevent selection bias and allow study participation for those of low socioeconomic status, the absence of technology literacy or access to a smartphone and/or computer or tablet is not an exclusion criterion. Participants in the App group who do not have technology access will be provided with a cell phone and a basic data plan to allow study participation. Individual participant timelines are illustrated in Figure 2. Transfer to adult care typically occurs at age 17 or 18 years at the participating centers.

### App development

The App was modified from the pre-existing MyREADY Transition BBD App,^14^ developed for youth with brain-based disabilities (BBD) by a Canadian team including coauthors (AHK, AV-DL, AJM).^15^ The BBD version of the App was developed in conjunction with a Patient and Family Advisory Council. Modifications to the BBD app to make it suitable for a CHD audience were led by a pediatric cardiologist (ASM), an ACHD cardiologist (AJM), a CHD psychologist (AHK), an information technology (IT) expert (AV-DL), and a Youth Advisory Council comprising 5 adolescents and young adults living with CHD. The Youth Advisory Council members were patients at 3 of the 4 participating sites and included both male and female patients. They met 3 times by video conference to provide feedback about the App; these meetings were facilitated by team members having experience leading young adult focus groups (GD, BA). Council members also provided feedback by email. Feedback addressed both the functionality and the content of the App. The council developed a list of frequently asked questions, which were included in the App, and advised on the format of video testimonials included in the App. Two Youth Advisory Council members took up roles as actors with lived experience of CHD, appearing in videos included in the App.

**Figure 3:**
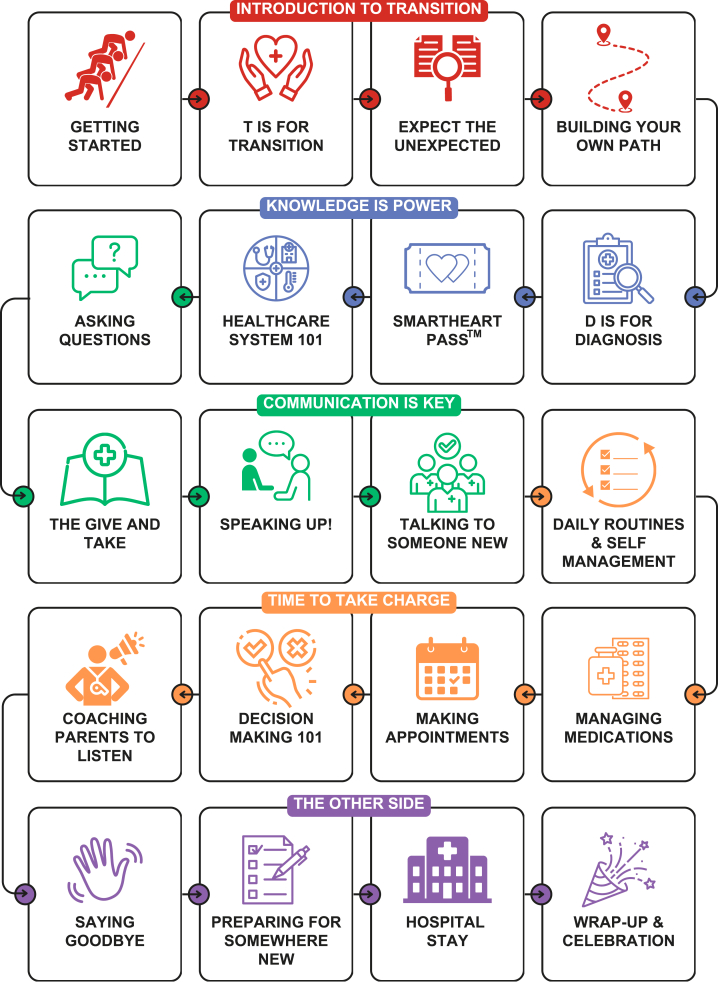
Content of the MyREADY Transition CHD App. CHD, congenital heart disease.

The App is compatible with iOS and Android phones, tablets, and computers and is available in both English and French. It has 20 modules (Fig. 3), each taking 10-15 minutes to complete. Content of the App includes but is not limited to (1) an introduction and definition of “transition” and “transfer,” (2) information about types of CHD, (3) a portable health summary with fields that participants can complete (SmartHeart Pass), and (4) tips on self-management and self-advocacy, including managing medications, making appointments, decision-making, and preparing for an elective hospital stay.

The App includes games, trophies, interactive quizzes, a “customizable mentor,” and other features meant to entertain and engage adolescents. There are 51 quizzes (true/false, multiple-choice answer formats) and 10 different types of games, including puzzles, “hangman”-style games, and find-the-object challenges. The mentor is a virtual “companion” that guides them through the App. The customizable nature of the App acknowledges diversity, as users can select a mentor that mirrors their preferences or own physical appearance. The mentor also has customizable accessories, with new ones unlocked as rewards for completing sessions. The time required for games, interactive quizzes, and customizing the mentor is integrated into the session content and included within the 10-15 minutes per module. However, users earn coins that they can spend on additional games in an arcade (game room) between sessions, if they wish. These optional games are not part of the curriculum.

The App content has been designed to reflect what our team has previously included in nurse-only teaching to enable a comparison between the *mode* of transition intervention rather than the *content* of the interventions.

### Intervention group (App + limited nurse time)

Participants in the App group meet once with a registered nurse (RN) during a regularly scheduled clinic visit, or in a virtual meeting if preferred by the participant, to (1) view an introductory video about the App, (2) download the App from the Apple or Android app store, (3) obtain a unique access code, (4) set up a password, (5) ensure that they can view the App, (6) receive information about how to obtain technical support, (7) learn about their cardiac anatomy and prior surgical and/or catheter interventions with the help of a diagram ± a heart model, and (8) review 3 potential future cardiac complications, as the App is not designed to provide information about a unique individual’s CHD. A checklist is used to ensure that all topics are discussed and App-related tasks completed. This checklist is an external metric being used for nurse documentation to make sure that all steps are completed, and not one that exists in the App. Participants are encouraged to complete 2-3 modules per week and all 20 modules within 3 months of enrollment; however, they have 18 months of access to the App. Participants in the intervention group will not have access to a cardiology nurse for transition teaching after the initial session.

### Control group (comprehensive nurse-only teaching)

Participants in the control group meet with an RN who is experienced working with teens for a single one-on-one session scheduled either during a routine cardiology clinic visit, or at an alternative time depending on the participant’s preference. If done on another date, the option of a virtual meeting is provided. Sessions are youth-oriented, interactive, and engaging. A PowerPoint presentation is used to facilitate teaching and ensure that all topics are covered. Teaching is conducted in either English or French, depending on the participant’s preference. Control group participants are not provided an access code to the App. Table 1 summarizes topics covered in both groups. An optional follow-up “refresher” teaching session is provided at the following cardiology clinic appointment (usually 12-24 months later) if desired by the participant.

**Table 1:** Content and mode of delivery of the intervention and control groups

No.	Content	Intervention group	Control group
1	Introduction to transition and its importance	App	RN
2	Provide handout of normal heart and explain blood flow and structures in simple terms	RN	RN
3	Diagram of the participant’s cardiac anatomy, including prior interventions and 3 potential future complications	RN	RN
4	Creation of a portable health summary, including name of CHD, prior cardiac interventions, and health care provider contact information	App	RN
5	Discussion of 3 potential future cardiac complications that are diagnosis specific and individualized	RN	RN
6	Medication management	App	RN
7	Discuss the concept of self-management	App	RN
8	3-sentence summary	App	RN
9	Mental health challenges—normalize and destigmatize	App	RN
10	Share resources∗	RN	RN

App and RN (registered nurse) refer to mode of delivery.

CHD, congenital heart disease.

∗ iHeartChange CHD transition website (iheartchange.org), Canadian Congenital Heart Alliance, Adult Congenital Heart Association, Vaping and Teens handout, Kids Help Phone website, and local mental health supports.

For participants in both groups, the RN reviews the participant’s medical record immediately before teaching sessions to become familiar with their cardiac history including CHD diagnoses, names and dates of cardiac surgical procedures and cardiac catheterizations, and current cardiac medications and doses.

### Group allocation

Participants are randomized by clusters defined by week of attendance in the pediatric cardiology clinic. As “week,” not study participant, is the unit of randomization, this is a cluster randomization design. This method of randomization (1) prevents 2 adolescents in the same waiting room being allocated to different groups and (2) facilitates scheduling of study RNs who are available at short notice to meet with participants. Cardiologists are not informed of group assignment, preventing potential bias by cardiologist cointervention. There is a 1:1 allocation of intervention:control weeks, stratified by site, using permuted blocks of randomly varying sizes of 2 and 4. We anticipate enrollment of 0-2 patients per week, that is, cluster size will be ≤2. To determine which weeks are intervention weeks vs control weeks, a biostatistician prepared a randomization sequence that is unique for each site. Allocation tables were locked and sent under password protection directly to a coordinator at each site.

### Treatment fidelity and quality assurance

Consistency between study RNs is a priority of this study for both the intervention and control groups. Team education has included virtual training meetings for study RNs affiliated with the intervention and control groups, held separately to avoid contamination of the intervention group or vice versa.

For both the intervention and control groups, the RN completes a checklist to document completion of each component as described in Table 1. Checklists are reviewed by team members (ASM, RNs) as part of ongoing quality assurance.

Audio recordings of sessions with study participants in the control group are conducted if the participant agrees. Evaluation of treatment fidelity is based on the review of nursing checklists and/or audiotapes by the lead author.

### Protecting against sources of bias

Participants, their parents, and the study RNs are aware of group allocation as blinding is not feasible. However, clinic support staff and pediatric cardiologists are not informed of group allocation. Participants are unaware of the primary and secondary outcomes and therefore unable to consciously influence the study findings.

### Primary outcome

The primary outcome is the change in the Transition Readiness Assessment Questionnaire version 5.0 (TRAQ) score, between baseline and 18 months. The TRAQ is the most rigorous transition readiness scale for adolescents.^16^ This 20-item scale has 5 subscales (Appointment Keeping, Tracking Health Issues, Managing Medications, Talking with Providers, and Managing Daily Activities). The overall scale has high reliability (Cronbach’s α 0.94). Item and mean scores range from 1 (low) to 5 (optimal).^17^ The TRAQ has a grade 5.7 reading level and takes approximately 5 minutes to complete. We have not observed a ceiling effect; the mean self-management score at baseline in the CHAPTER 2 study (16- to 17-year-olds) was 2.9 ± 0.8.^7^ All baseline questionnaires are completed in the cardiology clinic before meeting with a study RN.

### Secondary outcomes

Given the complex, multidimensional nature of the transition process, transition interventions must consider multiple outcomes. Therefore, several secondary outcomes are planned.

(1) Change in MyHeart scores between baseline, 1, 6, 12, and 18 months. The MyHeart scale (Supplemental Appendix S1) consists of 8 questions that assess participants’ knowledge of their cardiac condition. It is written at a grade 4.6 level and takes approximately 5 minutes to complete. MyHeart scores increase following nurse-only teaching.^6^, ^7^, ^8^
(2) Change in General Self-Efficacy (GSE) score^18^^,^^19^ between baseline and 18 months. The GSE is a 10-item questionnaire using a 4-point Likert scale that takes approximately 2 minutes to complete. Adolescents’ GSE scores predict multiple quality of life domains, including emotional, physical, and social.^19^ Team member Kovacs showed that among adults with CHD, lower GSE scores were associated with being female, unemployed, and having poorer functional class.^20^ Kovacs and McCrindle documented GSE scores in adolescents with moderate or complex CHD (n = 82); mean scores were 3.0 ± 0.6.^21^
(3) Excess time between pediatric and ACHD care, defined as the time interval in months between the final pediatric visit and the first adult visit, minus the recommended time interval between these visits. The “recommended time” interval was the interval suggested by the cardiologist at the final pediatric visit. For example, if the time between the final pediatric visit and first adult visit was 20 months but the pediatric cardiologist recommended 12 months, the excess time would be 8 months. Participants who have not attended the adult clinic by the end of the study period (ie, are “censored”) will still contribute to this outcome as survival analysis is designed to accommodate censored data. If the first ACHD visit takes place before the recommended time, the excess time will be zero. For the rare study participant not having a documented “recommended time,” recommendations for frequency of care will be taken from published guidelines.^22^^,^^23^ Our team has previously demonstrated the benefit of nurse-only teaching on this outcome measure.^7^
(4) Semistructured interviews of participants in both study arms are currently being conducted to understand participants’ experiences. For participants in the App group, additional inquiry is seeking a deeper understanding of the App’s usability and acceptability and its impact on quantitative outcomes. Interviews are conducted once, between 3 and 6 months after enrollment. Purposive sampling^24^ is used to ensure that participants in both arms of the study are selected, with representation of all 4 participating sites. Participants are invited to participate in a 30-minute semistructured interview by phone or video call. We anticipate conducting interviews with approximately 10 participants per study arm or until data saturation has been achieved and no new themes emerge. Interviewers are experienced, independent of intervention delivery, and follow a semistructured interview guide to ensure consistency (Supplemental Appendix S2). Participants in the App group are sent screenshots from the App as an optional review before or during the interview to remind them of specific aspects of the App (quizzes, puzzles, etc) that they will be asked about during the interview.

### Measurement of outcomes at follow-up

Participants are asked to complete the follow-up questionnaires independent of their parent(s). Adolescents who do not complete the follow-up questionnaires are contacted by mail, email, or text (depending on their preference) every 2 weeks for a total of 3 times, and then telephoned once, to be reminded. A CAD$50 gift certificate is provided to participants at each of the 1-, 6-, 12-, and 18-month time points to acknowledge their time and commitment to questionnaire completion. Documentation of first ACHD visit is sought during a medical record review 18 months after enrollment. If the first ACHD appointment has not happened at that time, documentation of the first ACHD appointment will be reassessed at study closeout.

### Proposed sample size

The sample size is based on the primary outcome, change in the TRAQ score. In our team’s CHAPTER 2 study,^7^ the mean difference from baseline of the TRAQ self-management score was 0.71 ± 0.90 at 18 months in the control group and 1.12 ± 0.86 in the intervention group (effect size 1.12 − 0.71 = 0.41). For the current study, the noninferiority margin between the intervention and control groups at 18 months has been conservatively set at 0.40. This represents less than half of a standard deviation. Anticipating the mean difference from baseline of the TRAQ self-management score of 1.12 ± 0.86 in the control (nurse-only) group (consistent with our CHAPTER 2 data) and estimating that a mean difference from the baseline in the App group will be no more than 0.40 less at 18 months (ie, 1.12 − 0.40 = 0.72), with 1-sided α = 0.025, standard deviation 0.8, and 80% power, we would require 64 patients per group (total 128). To account for clustering due to randomization by weeks with estimated average number of patients per cluster (per week) to be 2 and intraclass correlation coefficient = 0.1, the design effect is equal to 1.1, which increases the sample size to 71 per group (142 total). The CHAPTER 2 study had a dropout rate of 26% by 18 months.^7^ Assuming a 30% dropout rate, we aim to enroll 102 participants per group (204 total).

### Quantitative data analysis

Intention-to-treat analyses will be used, and all statistical tests will be 2-sided. Baseline characteristics of both groups will be summarized using descriptive statistics (eg, means, medians, standard deviations, frequencies, and proportions). Linear mixed models with random effects will be used to analyze the change in scores over time for each outcome variable. To account for the cluster randomization, randomization by 4 sites, and repeated individual values, effects of weeks (clusters), sites, and individual participant will be considered random. All outcome scores will be adjusted for the baseline in the models. To evaluate the effect of time on intervention, we will include an intervention group × time interaction in each model. Separate models will be developed for each outcome measure that include all data collection times of respective scores to assess treatment effects, with and without adjustment for baseline variables. Planned subgroup analyses are moderate vs complex CHD and by gender. Statistical analyses will be performed using SAS 9.4 (SAS Institute Inc, Cary, NC).

### Qualitative data analysis

Interviews will be transcribed verbatim, anonymized, and analyzed using reflective thematic analysis^25^ to identify and extract patterns in data about participants’ perceptions and views about the acceptability and usability of the App (intervention group), or acceptability and value of the nurse-only teaching (control group). Thematic analysis will be used inductively to investigate the barriers and facilitators to the uptake of the App by adolescents. Attention will be given to how gender and severity of CHD (moderate vs complex) influences participants' experiences including their engagement with and uptake of the App. Transcription and data analysis will occur concurrently to identify when new themes cease to emerge. To enhance the trustworthiness of the data analysis, 20% of the transcripts will be independently coded by Dimitropoulos, the lead qualitative researcher. To analyze the nursing logs and field notes for the control group, summative content analysis^26^ will be used to identify the frequency of the words employed and the potential meaning for each term to describe the participant’s interactions during the sessions with the nurse. Interviews, logs, and field notes will be analyzed independently by 2 team members and reviewed by the lead qualitative researcher. The qualitative research team will follow all steps to increase the validity, credibility, transferability, and dependability of findings by adhering to guidelines for publication of qualitative research studies.^27^

### Cost-effectiveness analysis

Total and incremental health care costs of providing app-based and nurse-only care will be determined. We anticipate that App-based care will have lower cost given the reduction in nursing time compared with standard nurse-only care, and if noninferior, determining total and incremental costs of providing App or nurse-only care will result in actionable results.

A microcosting approach will be used, comprising identification, measurement, and valuation of resources required for development and delivery of App-based or nurse-only care.^28^ This will include one-time costs (educational material and app development and design), intermittent costs (updated app and educational materials), and ongoing costs (IT costs, IT personnel, and health care professional time) and will be prospectively enumerated throughout the study. Valuation of resources will be based on current costs, for example, the wage rates of IT personnel and nurses. Annual per patient costs will be determined by estimating the scale and spread of one-time costs, frequency of intermittent costs, and capacity of health care providers (ie, how many patients can be provided transition care by one nurse), accounting for the size of participating programs. Sensitivity analysis will be conducted to account for scope and scale of programs (significant impact on one-time and intermittent costs) as well as a range of wage rates in various jurisdictions. We will use a similar approach to programmatic costing as our group has previously conducted.^29^

### Ethical considerations

The study has been approved by the Health Research Ethics Board at all participating sites. Written informed consent is obtained in all cases.

## Discussion

The READYorNot CHD study will rigorously evaluate a novel transition app compared with a previously established control^6^^,^^7^ (ie, nurse-only teaching) among older adolescents with moderate or complex CHD. The App content has been designed to meet the educational needs of adolescents with CHD.^30^ For example, given the high proportion of adolescents and young adults who experience gaps in care^31^, ^32^, ^33^ predisposing them to late cardiac complications,^34^ the App includes an emphasis that CHD is a lifelong condition that requires specialized care throughout adulthood. The App also addresses adolescent self-management skill development, including but not limited to medication management, appointment scheduling, development of a portable health summary (the SmartHeart Pass), and preparation for an elective hospital stay.

Li et al.^35^ recently published a systematic review of the usability and effectiveness of mobile health interventions, supporting transition in adolescents and young adults with chronic disease. Among 22 included studies, 18 (82%) involved a mobile app. Only 12 studies (55%) provided data on efficacy, and only 1 study was focused on the CHD population.^36^ The interventions ranged from simple text messages to complex interventions including games and engagement with health care providers. Six intervention themes were identified: medication monitoring and reminders, symptom tracking and monitoring, management goal setting, knowledge education and self-management skills training, incentives and reinforcement, and communication. Participants in these studies reported that apps were convenient, easy to use, and easy to access. However, some studies reported a decline in youth engagement with the app over time, as early as within 4 weeks.^36^^,^^37^ Adolescents and young adults preferred engaging elements such as visually appealing features, customization options, and entertainment functions, as well as disease-specific information, all of which were incorporated into the App. This review also reported that adolescents and young adults valued opportunities to communicate with their peers and health care providers. Although these features were not included, the App does include 1-way communication in the form of brief video messages of support from peers with affirming messages. This review demonstrated that overall, the evidence regarding the efficacy and long-term health benefits of these interventions is minimal, as most of the interventions were in the usability testing stage.

There is very limited literature with respect to transition app development in CHD. Lopez et al.^38^ previously developed an app for adolescents with CHD, with content that was informed by semistructured interviews of a large group of adolescents and young adults with CHD aged 15-22 years, parents, and 2 expert panels (3 adolescents per panel). Content includes a profile page with a diagram of the user’s native cardiac anatomy, a transition checklist, information on when to seek urgent medical care, the option for journal entries, and information about managing medications, answering doctors’ questions, and lifestyle topics including exercise, travel, and alcohol. Outcome data from this app have not yet been published. Han et al.^36^ conducted a randomized clinical trial of nurse-led transition teaching to evaluate if including a demonstration of 5 standard phone apps (Contacts, Calendar, Notes, Camera, and Reminders), currently available on iOS and Android phones, benefits adolescents’ self-management skills. The control group received the same teaching content, but without incorporating these apps. The purpose of encouraging youth to use these apps, collectively referred to as “Just TRAC it!” was to promote the development of their self-management skills.^36^ Transition readiness skills increased in both study groups, with no clear benefit for those in the “Just TRAC it!” group. However, most participants in the App group reported these 5 apps to be very or extremely useful, and by 6 months after enrollment, 100% of participants stated that they would recommend it to others.^36^

The development of the App was a resource-intensive process, requiring more than 12 months of content review by experts in CHD care and a Youth Advisory Council, despite the advantage of being able to develop the App on the platform of a pre-existing app for youth with BBD. However, if shown to be effective, the App has the potential to reduce dependence on nursing resources going forward, warranting a cost-effectiveness analysis. Nursing time and space in clinical settings can be barriers to delivering transition preparation for some programs. Furthermore, the App could reduce barriers imposed by social determinants of health. For example, living remotely from cardiac centers or having employment that does not enable time off can serve as barriers to attending cardiology appointments and reduce the likelihood of benefitting from nurse-only teaching. The App is also highly accessible; it will be continuously available to the intervention group participants for an 18-month period, allowing them to review the content repeatedly as needed. A racially and ethnically diverse study of adolescents and young adults with CHD reported that 94% had access to a mobile phone,^38^^,^^39^ and most individuals always keep their phone with them. In contrast, nurse-only teaching is a time-limited event, and although control group participants will have the opportunity to take home a portable health summary and diagram of their CHD, not all content will always be available to them.

### Potential limitations

Several threats to internal and external validity exist, which include the following: (1) cointervention: awareness of the study and of the need for health care transition may influence what cardiologists say to participants; however, both groups will benefit equally, and this will not create a bias toward one study group. Cardiologists will be unaware of group allocation, though they may inadvertently find out their patient’s group assignment. (2) Loss to follow-up: we anticipate that approximately 30% of participants will not complete questionnaires at all time points despite repeated reminders by text, email, or phone. A $25 gift card for completion of questionnaires at each time point was increased to $50 for participants in both study groups. When patients miss appointments, clinics will use all available contact information to arrange another appointment and keep patients in care. Participants who have not attended the ACHD clinic by the end of the study period will be censored and will still contribute to the secondary outcome of excess time to ACHD care. (3) App uptake: the App takes approximately 4 hours to get through, which is significantly more time-consuming than meeting with a nurse for an hour. Intervention group participants will be encouraged to do 2-3 modules per week, each taking 10-15 minutes. Videos, games, “trophies,” and other interactive features have been built into the App to keep users engaged. To encourage App completion, participants’ names are entered into a draw to win a pair of earbuds for each module they complete, which may bring into question real-world uptake of the App. Control-group participants are also entered into a draw for earbuds based on the number of questionnaires completed. (4) If there are delays in first adult clinic appointments resulting in excess time to ACHD care, reasons for such delays are unlikely to be well documented in the medical record and therefore unavailable to the study team. (5) The App group requires a brief teaching session with a nurse (Table 1), so access to the App is not independent of nurse teaching. However, App group nurses are different from “nurse-only” group nurses at all sites and receive separate training, to avoid contamination of the interventions.

In summary, the READYorNot CHD study is a multicenter, cluster randomized clinical trial comparing a novel transition app with limited nursing teaching to comprehensive nurse-only transition teaching for 16- to 17-year-olds with moderate or complex CHD. We are evaluating self-management skills, CHD knowledge, self-efficacy, time to first ACHD appointment, qualitative outcomes, and cost-effectiveness. The results of this study will inform patients, pediatric cardiology programs, and policy makers in judging whether this App provides clinically meaningful outcomes for adolescents and young adults living with CHD.
