# Supplementary material for: A Cluster Randomized Trial of an mHealth Intervention for Adolescents With Congenital Heart Disease: Rationale and Design of the READYorNot CHD Study
Source: CJC Pediatr Congenit Heart Dis. 2025 Jun 7;5(1):1–11. doi: 10.1016/j.cjcpc.2025.06.001 (PMC12946917; doi:10.1016/j.cjcpc.2025.06.001)
Supplement: Supplementary Material [file mmc1.pdf]

Supplemental Appendix S1:

CONSORT 2010 checklist of information to include when reporting a randomised trial*

Section/Topic	Item No	Checklist item	Reported on page No
**Title and abstract**			
	1a	Identification as a randomised trial in the title	1
	1b	Structured summary of trial design, methods, results, and conclusions (for specific guidance see CONSORT for abstracts)	2
**Introduction**			
Background and objectives	2a	Scientific background and explanation of rationale	4
	2b	Specific objectives or hypotheses	5
**Methods**			
Trial design	3a	Description of trial design (such as parallel, factorial) including allocation ratio	5
	3b	Important changes to methods after trial commencement (such as eligibility criteria), with reasons	N/A
Participants	4a	Eligibility criteria for participants	5
	4b	Settings and locations where the data were collected	5
Interventions	5	The interventions for each group with sufficient details to allow replication, including how and when they were actually administered	7-8
Outcomes	6a	Completely defined pre-specified primary and secondary outcome measures, including how and when they were assessed	9-11
	6b	Any changes to trial outcomes after the trial commenced, with reasons	N/A
Sample size	7a	How sample size was determined	12
	7b	When applicable, explanation of any interim analyses and stopping guidelines	N/A
Randomisation:			
Sequence generation	8a	Method used to generate the random allocation sequence	9
	8b	Type of randomisation; details of any restriction (such as blocking and block size)	9
Allocation concealment mechanism	9	Mechanism used to implement the random allocation sequence (such as sequentially numbered containers), describing any steps taken to conceal the sequence until interventions were assigned	9
Implementation	10	Who generated the random allocation sequence, who enrolled participants, and who assigned participants to interventions	9

Blinding	11a	If done, who was blinded after assignment to interventions (for example, participants, care providers, those assessing outcomes) and how	9
	11b	If relevant, description of the similarity of interventions	Table 1
Statistical methods	12a	Statistical methods used to compare groups for primary and secondary outcomes	12-14
	12b	Methods for additional analyses, such as subgroup analyses and adjusted analyses	12-14
**Results**			
Participant flow (a diagram is strongly recommended)	13a	For each group, the numbers of participants who were randomly assigned, received intended treatment, and were analysed for the primary outcome	N/A
	13b	For each group, losses and exclusions after randomisation, together with reasons	N/A
Recruitment	14a	Dates defining the periods of recruitment and follow-up	N/A
	14b	Why the trial ended or was stopped	N/A
Baseline data	15	A table showing baseline demographic and clinical characteristics for each group	N/A
Numbers analysed	16	For each group, number of participants (denominator) included in each analysis and whether the analysis was by original assigned groups	N/A
Outcomes and estimation	17a	For each primary and secondary outcome, results for each group, and the estimated effect size and its precision (such as 95% confidence interval)	N/A
	17b	For binary outcomes, presentation of both absolute and relative effect sizes is recommended	N/A
Ancillary analyses	18	Results of any other analyses performed, including subgroup analyses and adjusted analyses, distinguishing pre-specified from exploratory	N/A
Harms	19	All important harms or unintended effects in each group (for specific guidance see CONSORT for harms)	N/A
**Discussion**			
Limitations	20	Trial limitations, addressing sources of potential bias, imprecision, and, if relevant, multiplicity of analyses	17-18
Generalisability	21	Generalisability (external validity, applicability) of the trial findings	N/A
Interpretation	22	Interpretation consistent with results, balancing benefits and harms, and considering other relevant evidence	N/A
**Other information**			
Registration	23	Registration number and name of trial registry	2,5
Protocol	24	Where the full trial protocol can be accessed, if available	N/A
Funding	25	Sources of funding and other support (such as supply of drugs), role of funders	19

Citation: Schulz KF, Altman DG, Moher D, for the CONSORT Group. CONSORT 2010 Statement: updated guidelines for reporting parallel group randomised trials. BMC Medicine. 2010;8:18. © 2010 Schulz et al. This is an Open Access article distributed under the terms of the Creative Commons Attribution License (http://creativecommons.org/licenses/by/2.0), which permits unrestricted use, distribution, and reproduction in any medium, provided the original work is properly cited.

*We strongly recommend reading this statement in conjunction with the CONSORT 2010 Explanation and Elaboration for important clarifications on all the items. If relevant, we also recommend reading CONSORT extensions for cluster randomised trials, non-inferiority and equivalence trials, non-pharmacological treatments, herbal interventions, and pragmatic trials. Additional extensions are forthcoming: for those and for up-to-date references relevant to this checklist, see www.consort-statement.org.

Supplemental Appendix S2

READYorNot CHD
Participant Questionnaire #2
The MyHeart Scale

We would like to know how much you know about your heart condition. Your answers will help us provide services and education that are important for the transition to adult health care. There is no right or wrong answer and your answers will remain confidential and private.

Please complete these questions on your own, without your parent(s) input.

1. What is the name of your heart defect/condition? Be as specific as possible.

____

____

2a. Have you had any heart surgeries? ☐ (a) Yes ☐ (b) No ☐ (c) Not Sure

If YES, how many have you had? ______________________

If YES, what was the name of the operation(s), or what did the surgeon do? ____________

____

2b. Have you had any heart catheterizations? ☐ (a) Yes ☐ (b) No ☐ (c) Not Sure

If YES, how many have you had? ______________________

If YES, what were they for? ______________________

____

3. Could you explain your heart defect to someone else?

☐ (a) Not at all ☐ (b) A little ☐ (c) Yes

4. What are the medications you take for your heart, and what dose do you take?

☐ My heart medications are: ☐ I'm on no heart medications

1. ____________________ dose ____________________

2. ____________________ dose ____________________

3. ____________________ dose ____________________

4. ____________________ dose ____________________

5. ____________________ dose ____________________

5. What are the purposes/ reasons for your heart medications?

☐ Not applicable, I'm on no heart medications

☐ Reasons for my heart medications (in same order as above) are:

1. ______________________________

2. ______________________________

3. ______________________________

4. ______________________________

5. ______________________________

6. How long do you think you should be followed by a cardiologist who specializes in congenital heart disease?

☐ (a) When new problems arise ☐ (c) For the rest of my life

☐ (b) For a few more years ☐ (d) I don't know

7. Do you need to take an antibiotic before you see a dentist?

☐ (a) Yes ☐ (b) No ☐ (c) Don't Know

If 'Yes', why is this antibiotic recommended?

____

____

____

8. Did you get help with answering these questions?

☐ Yes, from: (indicate all that apply)

- ☐ the Internet
- ☐ a MyHealth passport
- ☐ pamphlets/brochures previously given to me about my heart
- ☐ Other (please specify):______________________________

☐ No, I answered these questions from memory alone

Supplemental Appendix S3

Ready or Not CHD

Qualitative Interview Guide

<u>Interview</u>

Instructions for Interview: Thank-you for taking the time to speak with us today. We are interested in learning about your experience with the [Transition App/nurse-led teaching session]. I'm going to ask questions about the [app/teaching session] and about how it may or may not have impacted you or your health. Your thoughts are really important to us to help us better understand the needs of young people with congenital heart disease. Don't worry if you don't have answers for everything, I'm here to help. It's ok to say that you don't know or ask me questions. Do you have any questions before we get started?

Are you in a place where you feel comfortable to talk with some privacy? If so, we can get started…

BEGIN AUDIO RECORDING SESSION

App Intervention Interview Guide:

1. I would like to start by talking about what it was like using the app. Can you start by telling me what you thought about the app in general?
    a. Probe: What was your first impression of the app?
    b. Probe (if used app over a period of time): What kept you coming back to use the App? Why do you think that was helpful/not helpful? Is there anything else that you think would be helpful to remember to use the app? Probe about reminders internal vs external to app.
    c. Probe (if app not used recently): You haven't used the app in a little while. Why is that? Is there anything stopping you from using it more? What are your reasons for not using the app more often?
    d. Probe for general and specific input on what aspects they liked more or less or neutral Can you think of something about the app that you liked? Disliked? Can you give me an example of what led you to like or dislike the App?
    e. Probe about how easy/difficult it was to use the app
    f. Probe about the appearance/flow of the app
    g. Probe about inclusion of games and quizzes in the app
    h. Probe about general idea of using an app, appearance, functionality

2. What information in the app was the most/least helpful to you?
    a. Probe about content: Was there a specific section of the app that you found more/less helpful (ie. Info about the adult hospital, about medication, about mental health)?
    b. Probe about different types of tasks: Was there a particular mini-game or challenge within the app that you found more/less helpful?
    c. Probe about how the intervention was helpful with them understanding the transfer of care to adult services, the transition to adulthood, their disease/diagnoses, medication, and navigating the health and mental health system.

3. Reflecting back on your experience with using the app, was there information about living with congenital heart disease that you didn't know before?

4. Reflecting back on your experience with using the app, what has changed (if anything) in your ability to manage congenital heart disease yourself?
    a. *If the participant responds that the intervention did not contribute to enhanced self-management, probe:* What could have been done differently (content, activities, psychoeducation info) to increase your ability to manage living with congenital heart disease?
    b. *If the participant responds that the intervention helped, probe:* Which modules/content/activities did you think were the most helpful in contributing to enhanced self-management of their congenital heart disease?

5. While using the app, you might have come across some information about self-advocacy. Self-advocacy means speaking up for yourself, making your own choices about your health/mental health care, knowing your rights and learning how to get information so that you can make informed decisions about your life. When you think about your experience of using the app, what (if any) self-advocacy skills did you learn? Probe about what modules/content/activities might have helped more/less with self-advocacy?

6. Is there anything else you would like to share about your experience using the app?

7. Do you think this app would help other young people with congenital heart disease? Why or why not?

8. Changing gears a little bit, I would like to ask about parts of your session with the nurse that *were not* about the app. Was there any other information or resources that the nurse gave you that you found useful? (Cardiac anatomy teaching, handouts, etc)
    a. Probe why/why not useful? Might it be information that you use in the future?
    b. Probe: Was there anything else that you wish the session had included?
    c. Probe: Other than the session with the nurse and the app, is there anything else that you think would be helpful to help you prepare for adult care?

9. In preparing for this conversation, what helped you remember the app? Was it the Ppt slides, the video, or something else?

10. As you might remember, in this study there were 2 different ways study participants could learn about transition to adult care: using the app, or a teaching session with the nurse. We've talked about your experience with the app, but I am interested to know if you think that one would be better than the other?

    a. What would make it better? In what way?

11. In the future, where do you think the research team should focus their efforts to help other young people with congenital heart disease learn about their health and the transition to adult care?

    a. Prompt: for example, should we focus on improving the app and making it available? Should we focus on improving the nurse session? Both? Do you have any other ideas about how we could help young people with congenital heart disease learn about these topics?

Thank-you so much for taking the time to speak with me today!

Nurse Intervention Interview Guide:

1. I would like to start by talking about your experience meeting with the nurse. Can you start by telling me what you thought about the nurse-led teaching session in general? *(If trouble remembering, prompt: The meeting with the nurse was the one where they talked about your heart anatomy, possible complications in the future, and medications. They might have talked about mental health, the effect of other drugs on your heart condition, and if you need medication before going to the dentist)*How would you describe your experience of the nurse-led teaching session?
    a. Was there a part of it that you enjoyed? Was there a part that you did not enjoy or that you would like to change?
    b. How did you feel going into the meeting vs after the meeting?
    c. Probe for general and specific input on what aspects they liked more or less or neutral
    d. Probe about the general idea of having a teaching session with a nurse?
    Probe about session content

2. What parts of the teaching session were the most/least helpful to you?
    a. Probe about content: Was there a specific topic that the nurse covered that you found more/less helpful?
    b. Probe about handouts and resources the nurse gave them.
    c. Probe about how the intervention was helpful with them understanding the transfer of care to adult services, the transition to adulthood, their disease/diagnoses, medication, and navigating the health and mental health system.

3. Reflecting back on your experience with the teaching session, what has changed (if any) in your ability to manage congenital heart disease yourself?
    a. *Keep in mind that participants may not have had a chance to use skills yet.* What management skills do you think you or other youth might use in the future?
    b. *If the participant responds that the intervention did not contribute to enhanced self-management, probe:* What could have been done differently (content, activities, psychoeducation info) to increase your ability to manage your congenital heart disease?
    c. *If the participant responds that the intervention helped, probe:* Which content/activities did you think were the most helpful in contributing to enhanced self-management of their congenital heart disease?

4. During the teaching session, you might have talked about self-advocacy. Self-advocacy means speaking up for yourself, making your own choices about your health/mental health care, knowing your rights and learning how to get information so that you can make informed decisions about your life. When you think about your experience of the teaching session, what (if any) advocacy skills did you learn? Probe about what content/activities might have helped more/less with self-advocacy.

a. *Keep in mind that participants may not have had a chance to use advocacy skills yet.* What self-advocacy skills do you think you might use in the future? Or probe what self-advocacy could youth use?

5. Do you think this teaching session would help other young people with congenital heart disease? Why or why not?

6. Is there anything else you would like to share about your experience meeting with the nurse?

7. As you might remember, in this study there were 2 different ways study participants could learn about transition to adult care: using the app, or a teaching session with the nurse. We've talked about your experience with the teaching session, but I am interested to know if you think that one would be better than the other?

   a. What would make it better? In what way?

8. In the future, where do you think the research team should focus their efforts to help other young people with congenital heart disease learn about their health and the transition to adult care?

   a. Prompt: for example, should we focus on improving the app and making it available? Should we focus on improving the nurse session? Both? Do you have any other ideas about how we could help young people with congenital heart disease learn about these topics?

Thank-you so much for taking the time to speak with me today!
